# Thinking Critically: How to Teach Translational Medicine

**DOI:** 10.3389/fpubh.2018.00284

**Published:** 2018-10-10

**Authors:** Richard G. Foty, Elizabeth M. Gibbs, Esther H. Lips, Madhvi Menon, Janet P. Hafler

**Affiliations:** ^1^Translational Research Program, Faculty of Medicine, University of Toronto, Toronto, ON, Canada; ^2^Department of Integrative Biology and Physiology, University of California, Los Angeles, Los Angeles, CA, United States; ^3^Antoni van Leeuwenhoek Hospital, Amsterdam, Netherlands; ^4^Brigham and Women's Hospital, Harvard Medical School, Boston, MA, United States; ^5^The Teaching and Learning Center, Yale School of Medicine, Yale University, New Haven, CT, United States

**Keywords:** education, curriculum design, translational medicine, hidden curriculum, communication, case study

## Abstract

Translational Medicine (TM) is a comparatively new field of study that focusses on the continuum of activities from the conception of an idea, to advanced clinical testing and the development of a new medical technology or drug. In recent years, graduate education programs have been established internationally to train a new generation of professionals with specific skills necessary to navigate the translational landscape. Literature in the area highlights the importance of integrating specific competencies relevant to translational medicine as part of curriculum development. In addition to developing a working understanding of core knowledge (e.g., ethics, funding, regulation, policy, etc.), skills including effective communication, reflection, interdisciplinary, and interprofessional collaboration are critical components of a skilled TM professional. Curriculum development must focus on content, while carefully selecting the teaching strategies that are most effective to achieve the desired outcomes, which is for learners to comprehend the complex material. The following publication presents a series of vignettes that describe the experiences of an associate professor of molecular biology, who is looking to explore her role in translational medicine and develop skills for an innovative approach to problem-solving. The vignettes are focused on a variety of teaching and learning strategies that can be used to teach translational medicine. Each vignette includes a description of the experience from the perspective of the learner and the faculty as it pertains to the teaching strategy, method of delivery, and learning outcomes. TM is as complex to teach as it is to learn. The specialized skills and knowledges that are part of the TM toolbox cannot all be taught in a lecture format. Educators must consider multiple strategies and select those which are most effective for achieving the learning outcomes.

## Background

Translational Research (TR) is a comparatively new field of study that focuses on the process of moving scientific knowledge into real-world impact. A subdivision of TR, Translational Medicine (TM) specifically encompasses the continuum of activities from the conception of an idea to advanced clinical testing and ultimately, to the development of new medical technologies or drugs. The definition of these terms has been evolving in the literature for over a decade (1).

## Competencies

Regardless of the definition, the ability to translate scientific knowledge into real-world health impact requires specialized core knowledges (Biomedical research, intellectual property, funding, regulation, legal issues, ethical issues, preclinical testing, design of preclinical, and clinical trials) and skills (networking, team-building, strategic thinking, creative problem-solving) (2). In addition to other programs in translational medicine, we have identified persuasive communication, as well as interdisciplinary and extraprofessional collaboration, as core skills. In recent years, graduate education programs have been established internationally to train a new generation of professionals with these specific skills necessary to navigate the translational landscape. Many teaching strategies have been proposed and employed to deliver this content. However, there is no consensus regarding the best methods of instruction.

Curriculum development in medical education is a process that combines educational theory and methodology with specific content, then evaluates its impact (3). The process equally focuses on content and the most effective teaching strategies for the learners to comprehend this complex material. Faculty in TM education programs are often recruited from academic medical centers for their content expertise and experience in delivering health care. However, they may not be trained in the competencies necessary for effective teaching, or curriculum development.

The pedagogical framework developed by Thomas (4) includes six essential steps to curriculum design in medical education: Step 1: Identify a problem; Step 2: Examine the particular needs of the audience; Step 3: Develop goals and measurable learning objectives; Step 4: Choose the educational strategies; Step 5: Devise steps for implementation, and; Step 6: Consider evaluation and feedback. The framework is a dynamic, interactive and systematic process, but do not always follow each other in sequence (3). One of our authors collaborated to create a curricular design based on Schwab (5) which focused on how we think critically about education and how we aim to teach TM. This design includes several educational strategies that integrate the hidden and the formal curriculum, and is a principle based curricula [Table 1; (6)]. The pedagogy is based on the constructivist learning theory (7) so that each participant has the opportunity to develop their own knowledge base in TM. Both large and small group case-based learning sessions are integrated into the curriculum along with short lectures, team-building and collaboration, recording of presentations, self-assessment, and mentoring groups that include mentoring from peers and expert faculty members.

**Table 1:** Chart of teaching strategies.

**Strategy**	**Advantages**	**Disadvantages**	**Example situation**
Lectures/Presentations	Good for primary explanation and clarifying concepts	Teacher-centerd, not learner-centerd; Generally, cannot review the presentation	Review of the Translational Medicine pathway
Case discussion	Useful for developing: problem-solving; critical thinking; demonstrating different points of view; effective communication; teamwork	May take time for the concepts to evolve; some members of the group may not participate	Case study to identify unmet patient needs; understand underlying problems; brainstorm possible solutions and implementation strategies
Questioning	Broadens/challenges ideas; involves the learner in the process	Skills are required to understand the range of question types	What is the participants knowledge of Translational Medicine?
Practicing	Begins to change behavior with personalized instruction; reinforces concepts	Takes time; may require observation by an instructor	Presentation workshop.
Feedback	Begins to change behavior; essential for learning	The teacher may not give useful or even any feedback	Peer review of presentation
Handouts/printed materials	Often used to illustrate initially and then useful for later reference	Information may not convey nuances; quantity of information may overwhelm	Graphic overview of Translational Medicine pathway
Computer-assisted instruction: e-learning	Good for initial instruction; practice; repetitions, and; future reference	The learner may need to obtain basic computer skills before using, may have mechanical" quality	Online materials required to establish a base knowledge of the day's topic before a seminar
Simulated cases/role play	Useful in helping learner apply material	Learners may feel threatened; may be difficult to relate to the character or situation	Practicing an elevator pitch; how to present to a chair or a foundation to obtain funding; Feedback is given
Video recordings	Useful in support of content presented in a lecture	Need audiovisual equipment, may be difficult to relate to the character or situation	A patient and family perspective
Slides	Visual reinforcement good for clarity; useful when presenting complex material	Information is very brief, cannot easily repeat the information	Introductory lecture on intellectual property law
Reading	Good for instruction, future reference, further exploration	No interaction with people	Reading in preparation, or as a follow-up to in-class discussion
Review, repetition	Reinforces concepts learned	Time-consuming	Having teams iterate on a conceptual prototype to achieve a solution to a patient need
Reflection	Examines aspects of an experience and develops reflective practice skills; allows expression and determines meaning	Time-consuming	Morning debrief sessions and journal writing

## Rationale

**Figure 1:**
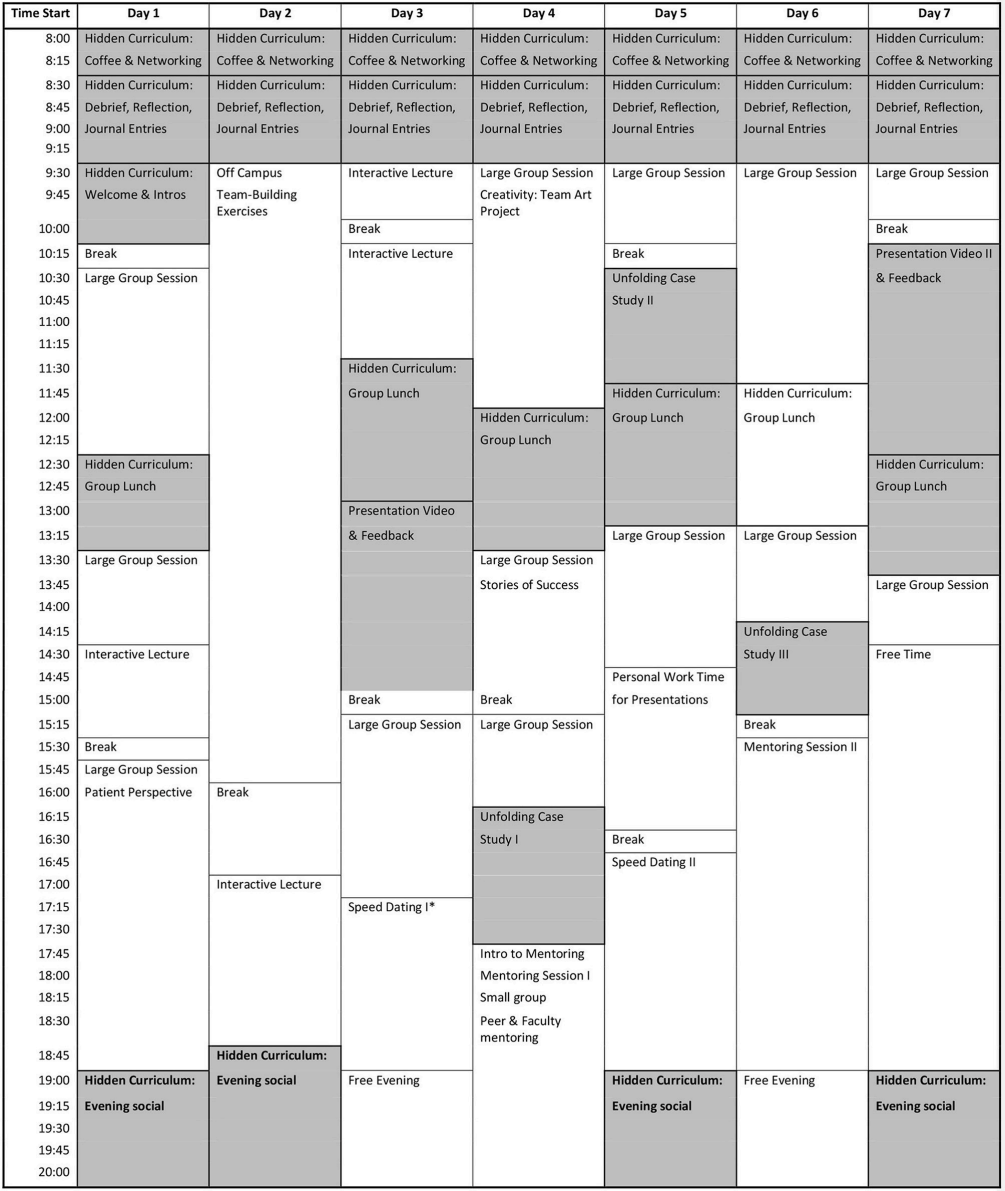
Sample curriculum design for translational medicine. Shaded area indicates strategies that are highlighted in this publication. *Participants have the opportunity to voluntarily sign up and meet individually with a faculty member for 10 min.

The innovative curricular design (Figure 1) presented below is aimed at teaching scientists and leaders who are working or intending to work, in the field of TM. In this article, we focus specifically on Step 4 of Kern's Model which addresses our educational objectives, our material, and our audience. We designed the curriculum to: (1) Analyze the business, scientific and regulatory aspects of TM; (2) Explore the challenges professionals encounter in TM including how to teach and learn; (3) Develop critical thinking skills to approach the challenges in TM, and; (4) Develop communication skills for presenting various topics to a broad spectrum of learners.

The skill of thinking critically in an open and safe learning environment is paramount in the curriculum as we focus on teaching to promote learning. We selected teaching strategies that enable students to master content using critical thinking skills. We designed the curriculum to teach material by enhancing the learners' ability to think and engage in their own learning, collaborate, and learn together.

## Learning objectives

The objectives of this article are to describe an innovative curriculum designed to teach TM, however, it could also be generalized to train other health professions. The following presents a series of vignettes that describe the experiences of a learner Susan Dias, Ph.D. Susan is an associate professor in molecular biology, who is attending a certificate program in TM as her first exposure to the discipline. She is a primary investigator within a hospital setting and has become increasingly frustrated. She is unsure that patient needs are driving her scientific questions, and she believes her narrow hypothesis-driven research is limited. Susan is looking for a different, more innovative approach to problem-solving. The vignettes are focused on a variety of teaching and learning strategies and theories that can be used to teach translational medicine and include case-study design, presentation skills, reflection, team-building, and the hidden curriculum. In addition to these vignettes, each section below will include key points for faculty to consider as it pertains to the respective teaching strategy, learning outcomes, and a personal reflection. All persons and data presented in this manuscript are fictional.

## Part I-the hidden curriculum (Table 2)

It's the morning of my first day at the certificate course in TM. I'm excited, albeit a little anxious to meet the other attendees and get started. The foyer of the building is filled with nervous energy; the room full is of people sipping espresso and making small talk. I grab a coffee just as we're asked to make our way into the lecture hall. As I walk through the doors, I'm taken aback. The room is less a lecture hall than a large dining room with 30 chairs arranged in a circle and no lectern or obvious indication of which way we should be facing. Intrigued and curious, I take a seat. When the room settles, the person seated to my right who I now realize is leading the session, welcomes us to the program. We are each given a notebook to use as our journal through this experience. In them, we are asked to write our reflection on each day's activities which will be used to frame an open discussion each morning in a debrief meeting with a faculty member. I'm intrigued by this reflection exercise. What exactly is it that they want us to write in our journals?

**Table 2:** Key points of the hidden curriculum.

Create a safe environment (team-building activities) Intentional room setup (e.g., the orientation of chairs, the absence of computers or projectors) Use multiple approaches to influence using the hidden curriculum (8) ° Formally structured and intended social activities ° Informal, unplanned, and unscripted social activities ° Less tangible influences such as organizational culture ° Debrief and journal entry

A flip chart is brought out, and we are each asked in turn to write down our names and tell an associated story. I'm already feeling a little nervous, although it has only been a few minutes. We spend quite a bit of time on these introductions, which actually turn out to be a fantastic ice-breaker. Something about seeing the names written down and associating them with a story made it very easy to remember everyone.

The next team-building activity is even more unusual. We stand up and are asked to converse with five different attendees, beginning each time with a different question: (1) Who are you? (Do this without discussing your name, job position, educational background, roles in life, and place of stay or birth); (2) Why did you start working where you work and why do you work here today? (3) What frustrates you the most about your work (concerning translational medicine)? (4) What gives you energy and fuels your engine through the day? (5) What is something small, or more significant you would like to change in your work environment concerning translational medicine? (9).

I hesitate. Discussing my passions, frustrations, and aspirations with a group of individuals I've just met is even more uncomfortable than the introductory exercise. Timidly, I approach one of the attendees, then another. I quickly realize that this is a very diverse group of people in different stages of life, from different backgrounds, disciplines, and professions. Despite these differences, I feel myself connecting with my new peers. We all share common challenges and frustrations; we are looking for ways to have a more positive and direct impact on patient health.

After a full day of sessions, the program has us head to a nearby restaurant for a dinner and social. Faculty and attendees are talking about the day, and our family lives back home. We're enjoying the night so much we hardly realize how late it is. Knowing we have an early morning ahead of us, we say our goodnights and retire for the evening.

### Reflection and journal entry

It's my first day and I'm starting to feel a sense of connection with the other participants and the faculty. The morning introduction exercises and the informal interaction at dinner pushed me far outside my comfort zone. I registered in the program because I felt I needed a fresh approach to research. Maybe this is where it starts, and these activities are meant to create a safe environment where we feel comfortable to share, grow, and learn from each other. I wonder why this has not worked as effectively at other conferences or courses I have attended? I imagine it has something to do with these well-programmed interactions and having been taken away from our daily routines. This purposeful setup has most certainly contributed to building this fantastic network of new colleagues.

This must be the kind of reflection they're expecting of me. Sitting down in silence for 10 min to pen down my thoughts is refreshing. This is definitely something I am going to incorporate in my life back home.

## Part II–the unfolding case (Table 3)

The next morning starts similarly with an early morning social over coffee. Groups of six participants are assigned to separate classrooms where we are greeted by two faculty members. This is the unfolding case, and the faculty members are to serve as facilitators to guide us.

**Table 3:** Key points of the unfolding case.

Occurs over 2 or more sessions Small groups of 4-6 people are ideal to have all participate. The group members should have diverse expertise (interdisciplinary, interprofessional) complementary skills sets. Role of the Facilitator(s): Actively listen to the discussion to ensure the learning objectives are being met Allow learners to set the agenda If all the group members share similar opinions or arrive at a consensus too quickly, ask questions that require the group to approach the problem from different perspectives. Encourage the group to voice conflicting viewpoints and support their own view. A good case: (10–12) Tells a story Has a dilemma to be solved Is relevant to the reader Is real rather than fabricated Specific to TM, a good case also: Requires knowledge from multiple disciplines and professions Has no single correct answer, but several paths to follow that may lead to a positive outcome Requires learners to view problems from multiple perspectives Draws on skills and knowledges that have been previously introduced, or Is designed to introduce new skills and knowledges Has specific learning objectives (e.g., Critical thinking, creative thinking, collaboration, effective communication)

We are presented with a case study on finding biomarkers for a new cancer drug that reads like a story. There is a protagonist, several supporting characters, a clear plot line, and it's actually quite engaging. The dilemma presented feels like a puzzle that I really want to solve.

The facilitators sit silently at the table, while we begin our discussion. Within minutes our entire group is speaking over each other, and each of us is offering a different opinion; each of us insistent that ours is the best way forward. After several minutes of this cacophony, I ask the facilitators if we are on the right track. They smile and suggest we begin by ensuring that everyone in the group understands the terminology in the case, and precisely what the question or dilemma is. Assuming we all understand the topic seems to be our first mistake. Once we reviewed the case and all understood the tasks at hand, the facilitators suggest we spend a few minutes familiarizing ourselves with each other's professional expertise. Amongst the other participants, there is a basic scientist, an epidemiologist and someone who works in marketing for pharmaceutical companies. Like me, these three participants have never seen a patient professionally. The final two participants are physician researchers involved in clinical trials.

Having a better handle on the various skills we had around the table, we begin to brainstorm the different angles of the problem. As a lab scientist, my thoughts immediately swing to the *in vitro* and *in vivo* experiments that must be done to better understand the biologic question. However, I soon realize that there is much more at stake and that the lab component is merely one tiny slice of a much larger pie.

The case unfolds over three separate sessions. While I'm able to contribute to the conversation at the outset, I find myself reserved and quiet when the group begins to discuss issues around intellectual property, funding, clinical trials, patient involvement, companion diagnostics, ethics, and regulation. This case is much more complex than I had initially thought and I'm thankful we have so much diverse expertise sitting around the table.

### Reflection and journal entry

When we started the case study, I thought the way forward was quite evident. The dilemma was rooted in basic science, and while I am well equipped to contribute to the problem-solving, I wondered what value an epidemiologist and marketing wizard might add to the discussion? Evidently, I was short-sighted. If I had approached the problem in my typical fashion, I would have missed many of the critical points raised by the other members of my group. I've never contemplated the importance of co-creating a research program with patients.

When we first started our discussion, each group member was sure they knew the correct answer. We jumped to solutions without ensuring we all understood the problem. We had failed to listen and engage each other. We weren't drawing on the collective skills sets we had around the table. I was also acutely aware of the amount of space each participant took up in the conversation. One participant, in particular, dominated most of the discussion. He presented many ideas and often interrupted others. I, on the other hand, tended to be more reserved with my comments, especially when the discussion moved into content areas in which I was less familiar. So much new information was thrown at me that I didn't have the chance to absorb and think critically. My mind was getting overloaded, and I was struggling to keep up. In retrospect, the suggestions made by the faculty to get us on track were so simple. I'm always in such a hurry to get to the answer; I should really spend more time contemplating the question.

I also realize translation requires a breadth of knowledge and many different skills. While I'm an expert in my field, there's just so much more I need to know to effectively translate my discoveries and improve the health of patients. I would have never been able to get through this case study without the other members of my group. Maybe it's time I start collaborating with my colleagues, rather than competing with them?

## Part III–communication skills (Table 4)

I was dreading the presentation skills session all week. While I often present my research to different audiences, I wasn't eager to have my presentation video recorded and scrutinized by the group. The night before the session, I spent about an hour preparing my 3-min talk: a short research pitch to a funding agency.

**Table 4:** Key points of the presentation feedback.

Phase 1 - Encourage students to spend time planning: Know the purpose of your talk Know your audience Tell a story Be clear about the take-home messages Nervousness is normal. Practice! Practice! Practice! Phase 2 - The exercise: Create a safe environment to practice skills and receive constructive feedback from peers Give the talk Record the talk Watch the talk privately and self-assess (students are often their own worst critic) Phase 3 - Feedback: Share your self-assessment Receive peer and faculty feedback

I stand in front of a small group of five participants. The faculty facilitator sets a laptop in front of me to record my presentation. My anxiety is increasing. I had never seen a recording of one of my presentations, and I feel self-conscious and uncomfortably aware of every aspect of my speech. Was I talking too quickly? Was I moving too much? Is anyone even interested?

At last, I finish the presentation. I'm asked to review the recording in the adjoining room while the group discusses my talk in detail. As I press play, I'm wondering who this person is on the screen. Watching myself immediately after having given the talk is startling, and it is clear to me that I could have been much smoother. I notice some nervous habits in my movements, and I can hear the hesitation in my voice throughout the presentation.

I make some notes and return to the group. The facilitator asks how I felt that it had gone. I share my observations and overall negative feelings about the presentation. To my surprise, no one feels that I had hesitated or paused too much during the talk, but they do mention my frequent use of “filler” words. They also suggest I work on eye contact with the group while presenting. I hadn't considered this during my own-self assessment, but I realize that I can quickly get caught up in my slides and not focus on the audience.

### Reflection and journal entry

The communication exercise wasn't nearly as terrifying as I had feared. Having been videotaped was tremendously useful. I don't usually take the time to evaluate my presentations, and I rarely get direct feedback from my colleagues. As it turns out I'm not nearly as bad at presentations as I had thought, though the group did point out some nervous habits of mine like my use of filler words. I hadn't noticed this before, but as soon as it was pointed out, I realize that I could make my narrative sound smoother and more confident by eliminating words and phrases that don't add any value to the talk. I really need to work on that. Overall, the exercise was very valuable and far more helpful than I expected. I'm already feeling more confident, and I know the comments I received will strengthen my delivery. We're being given the opportunity to incorporate the feedback we received and present again to the group tomorrow. Time to start practicing!

## Discussion

In this publication, we have presented selected teaching strategies that are important in the instruction of TM. The above sections include key points for faculty to consider when using each strategy as well as vignettes that describe the experience from the learner's viewpoint. First, the hidden curriculum is designed to create a safe environment in which to build teams. The faculty gently pushed learners out of their comfort zones in many of the teaching strategies, to challenge them to have new experiences and examine these experiences from different perspectives. Second, the unfolding case uses active learning to engage students. The participants are challenged to learn from their own knowledge base—collaborate with the group to prioritize what is essential to learn, rather than relying on the faculty to indicate what is important. Effective case-based learning requires learners to communicate clearly, think critically and creatively and function as a team, using the strengths of the individual members to inform the best way forward. Third, persuasive communication is an essential part of the TM toolbox. Again, however, it has been historically overlooked in scientific education. In the lab, in the clinic, in grant applications, ethics reviews and even in front of the media, it is crucial for scientists to be able to communicate clearly and succinctly in such a way that is accessible to their audience.

The common thread between each of these strategies is critical reflection. While content may be delivered using an appropriate strategy, the more profound learning opportunity lies in the learner's ability to reflect on how they experienced the curriculum and discover the relevance and importance of each activity. In the vignettes above, Susan was first asked to write her reflections on the day's events in her journal. The following day begins with a group discussion around the individual reflections. Discussing the experiences and reflections of our peers can help provide insights from different points of view and may aid in a more profound understanding of the experience.

In 2004, Ash and Clayton (13) developed the DEAL model for critical reflection. The model contains three steps: (1) Describe the experience in an objective and detailed manner; (2) Examine the experience as they relate to specific learning outcomes (e.g., personal growth, team dynamics, patient engagement) and; (3) Articulate the Learning and how it will be used in the future. A detailed and objective description of the experience gives the learner a firm foundation on which to look for meaning. Learners often overlook this step and choose instead to start the interpretation process immediately. In doing so, they may miss critical details of the experience. For instance, while it is important to know where the experience took place, who was there and what they did, it is equally important to ask who wasn't there and what didn't they do. In examining the experiences, the learners begin searching for meaning. This step is linked directly to the learning outcomes of the exercise. For example, if the goal is to reflect on a conflict that arose within a team, one may ask: What was the cause of the conflict? What was the trigger? What were the perspectives of each individual involved? How did the other party interpret those perspectives? It is also essential for the learner to be able to articulate what they have learned. The articulation should be actionable, such that it will provide further guidance to deepen and improve the quality of their learning and their future actions. This step consists of four prompts to guide the learner: (1) What did you learn? (2) How did you learn it? (3)Why does it matter? (4) What will you do in light of it?

The specialized skills and knowledges considered to be part of the TM toolbox cannot all be taught in a lecture format. Instead, educators must think carefully about which strategies are most effective for the anticipated outcomes. In Figure 1 we present a curriculum which we designed for a 7-day TM program, but the curriculum could be modified to take place over a longer period of time. The curriculum includes several strategies in addition to the few we have highlighted in this publication. Design of this curriculum followed Kern's model and is the result of several years of an effective PDSA assessment theory approach of Plan, Do, Study, Act. TM is as complex to teach as it is to learn. Selecting the most effective teaching strategies that are carefully placed in a well-designed curriculum is key to achieving one's intended outcomes.

## Author contributions

RF wrote the introduction and discussion sections, consolidated the remaining sections and was responsible for the final editing. MM wrote the section on the hidden curriculum and contributed to manuscript review and editing. EL wrote the section on the case discussion and contributed to manuscript review and editing. EG wrote the section on communication and contributed to manuscript review and editing. JH contributed to the conceptualization of the paper, the introduction, discussion, table creation, manuscript review and editing.

### Conflict of interest statement

The authors declare that the research was conducted in the absence of any commercial or financial relationships that could be construed as a potential conflict of interest.
